# Osteogenic Matrix Cell Sheets Facilitate Osteogenesis in Irradiated Rat Bone

**DOI:** 10.1155/2015/629168

**Published:** 2015-05-12

**Authors:** Yoshinobu Uchihara, Manabu Akahane, Takamasa Shimizu, Tomoyuki Ueha, Yusuke Morita, Shintaro Nakasaki, Tomohiko Kura, Yasuaki Tohma, Akira Kido, Kenji Kawate, Yasuhito Tanaka

**Affiliations:** ^1^Department of Orthopedic Surgery, Nara Medical University, 840 Shijo-cho, Kashihara, Nara 634-8522, Japan; ^2^Department of Public Health, Health Management and Policy, Nara Medical University School of Medicine, 840 Shijo-cho, Kashihara, Nara 634-8521, Japan; ^3^Department of Biomedical Engineering, Doshisha University, 1-3 Tatara Miyakodani, Kyotanabe, Kyoto 610-0394, Japan; ^4^Department of Orthopedic Surgery, Nara Medical Center, 2-789 Shichijo, Nara, Nara 630-8053, Japan; ^5^Department of Arthroplasty and Regenerative Medicine, Nara Medical University, 840 Shijo-cho, Kashihara, Nara 634-8522, Japan

## Abstract

Reconstruction of large bone defects after resection of malignant musculoskeletal tumors is a significant challenge in orthopedic surgery. Extracorporeal autogenous irradiated bone grafting is a treatment option for bone reconstruction. However, nonunion often occurs because the osteogenic capacity is lost by irradiation. In the present study, we established an autogenous irradiated bone graft model in the rat femur to assess whether osteogenic matrix cell sheets improve osteogenesis of the irradiated bone. Osteogenic matrix cell sheets were prepared from bone marrow-derived stromal cells and co-transplanted with irradiated bone. X-ray images at 4 weeks after transplantation showed bridging callus formation around the irradiated bone. Micro-computed tomography images at 12 weeks postoperatively showed abundant callus formation in the whole circumference of the irradiated bone. Histology showed bone union between the irradiated bone and host femur. Mechanical testing showed that the failure force at the irradiated bone site was significantly higher than in the control group. Our study indicates that osteogenic matrix cell sheet transplantation might be a powerful method to facilitate osteogenesis in irradiated bones, which may become a treatment option for reconstruction of bone defects after resection of malignant musculoskeletal tumors.

## 1. Introduction

Reconstruction of large bone defects after malignant musculoskeletal tumor resection is a significant challenge in orthopedic surgery. Several procedures have been attempted for reconstruction of such bone defects, including conventional autogenous bone grafting [1], vascularized bone transplantation [2], allogeneic bone grafting [3], and prosthetic replacement [4]. Although prosthetic replacement is one of the standard procedures for limb salvage operations, loosening, breakage, and wear particle formation of the prosthesis can cause clinical problems [4, 5]. Allografting is still uncommon in some Asian countries, especially in Japan, for socioreligious reasons.

Recently, intraoperative extracorporeal autogenous irradiated devitalized bone grafting has become a treatment option for reconstruction of bone defects after malignant musculoskeletal tumor resection [6, 7]. Surgeons can reconstruct the bone defect with the autogenous irradiated bone, resulting in an ideal shape and functional reconstruction because the bone maintains its original shape with tendons or ligamentous tissues attached to their original sites. Moreover, this method does not require harvesting bone from healthy sites, such as the pelvis, and is less problematic than allografts that might result in virus transmission and/or immunoreactions. However, there are some issues with this method. For example, the osteogenic capacity is completely lost by irradiation and nonunion or delayed union often occurs [5, 8]. Therefore, a novel method is required to facilitate osteogenesis for bone defect reconstruction by autogenous irradiated (devitalized) bone grafts.

We previously reported a cell transplantation method in which bone marrow-derived stromal cells (BMSCs) are cultured and lifted as a cell sheet structure. The cell sheet has an osteogenic potential and is therefore designated as an osteogenic matrix cell sheet [9, 10]. Osteogenic matrix cell sheets can be freely transplanted onto a scaffold at a nonunion site and enhance bone union with bridging bone formation around the nonunion site [11]. This finding indicates that the osteogenic matrix cell sheets may become an osteogenic cell source for autogenous irradiated devitalized bone grafts.

In the present study, we established bone defects in the femurs of rats, which were reconstructed using autogenous irradiated bone grafts together with transplantation of an osteogenic matrix cell sheet. The aim of the present study was to evaluate whether the osteogenic matrix cell sheet enhances bone union in such a model.

## 2. Materials and Methods

### 2.1. Animals

The experimental procedures were approved by the Animal Experimental Review Board of Nara Medical University. Fisher 344 rats were purchased from Japan SLC (Shizuoka, Japan). Seven- and 12-week-old male rats were used to prepare bone marrow cells and establish the autogenous bone graft model, respectively.

### 2.2. Preparation of Bone Marrow Cells

The method for BMSC preparation has been reported previously [9, 11]. Briefly, bone marrow cells were obtained by flushing out the femur shafts of 7-week-old male Fisher 344 rats with 10 mL culture medium. The cells were collected in two 75-cm^2^ culture flasks (Corning, NY, USA) containing 15 mL minimal essential medium (Nacalai Tesque, Kyoto, Japan) with 15% fetal bovine serum (Gibco, Life Technologies, Carlsbad, CA, USA) and antibiotics (100 U/mL penicillin and 100 *μ*g/mL streptomycin; Nacalai Tesque). The cells were cultured in an incubator at 37°C with 5% CO_2_. At confluency, the primary cultured cells were trypsinized from the flasks using trypsin/EDTA (Nacalai Tesque).

### 2.3. Preparation of Osteogenic Matrix Cell Sheets

**Figure 1:**
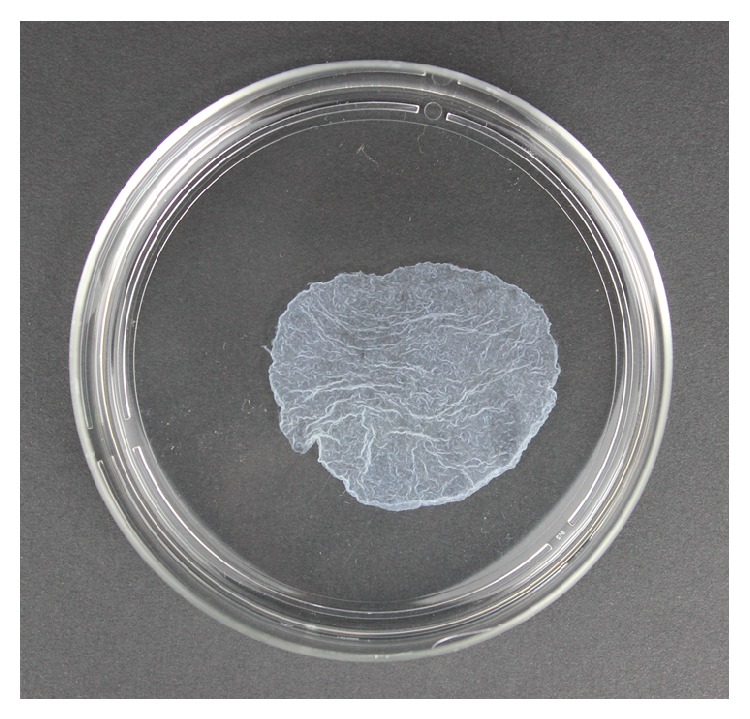
Macroscopic appearance of an osteogenic matrix cell sheet.

We have previously reported the method for osteogenic matrix cell sheet preparation [9]. Briefly, the trypsinized BMSCs were seeded at a density of 1 × 10^4^ cells/cm^2^ in 10 cm dishes (100 × 20 mm; Corning) containing medium with 10 nM dexamethasone (Dex; Sigma, St. Louis, MO, USA) and ascorbic acid phosphate (Asap; L-ascorbic acid phosphate magnesium salt n-hydrate, 82 *μ*g/mL; Wako Pure Chemical Industries, Kyoto, Japan) and cultured to confluency (approximately 12–14 days). The cells were rinsed twice with phosphate-buffered saline (Gibco, Life Technologies), and then the osteogenic matrix cell sheet was lifted by a scraper (Figure 1).

### 2.4. Preparation of Devitalized Bone Grafts

Femurs were obtained from 12-week-old male Fisher 344 rats. A column-shaped bone fragment of 10 mm in length was cut from the diaphysis of the femur using a bone micro-saw. It has been reported that tumor cells can be sufficiently devitalized by >50 Gy [5, 14]. Therefore, irradiation by a single exposure to 60 Gy (irradiation conditions: X-ray tube voltage, 125 kV; X-ray tube current, 20 mA; filter, 0.5 Al and 0.1 Cu) was performed to prepare the devitalized bone.

### 2.5. Transplantation of an Osteogenic Matrix Cell Sheet with an Autogenous Irradiated Bone Graft

**Figure 2:**
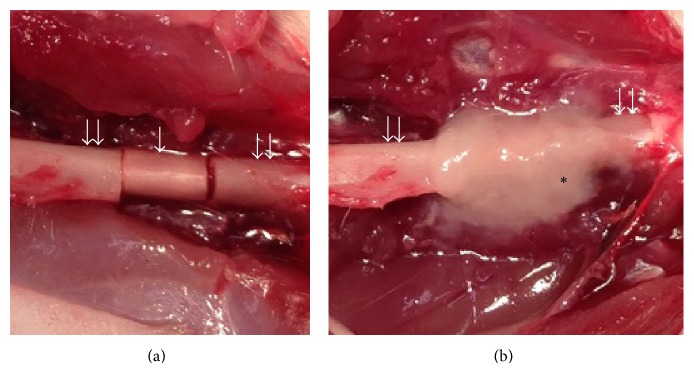
Osteogenic matrix cell sheet transplantation with an autogenous irradiated bone graft. Single and double arrows indicate grafted bone (irradiated bone) and the host femur, respectively. Asterisk indicates osteogenic matrix cell sheets. (a) Irradiated bone was fixed to the host femur using Kirschner wire. (b) Two osteogenic matrix cell sheets were wrapped around the grafted bone without suturing.

An autogenous devitalized bone graft model was established in the femur of 12-week-old male Fisher 344 rats under anesthesia. Briefly, a lateral incision was made on the right hind limb and the vastus muscle was divided longitudinally to expose the right femur. The center of the femur shaft was cut and a 10 mm length of bone was removed to establish a bone defect. The bone defect was replaced with a previously prepared devitalized bone graft. The devitalized bone graft was fixed to the host femur using Kirschner wire (1.2 mm diameter), which was inserted into the intramedullary femur shaft from the distal femur condyle in a retrograde fashion. Subsequently, two osteogenic matrix cell sheets were wrapped around the grafted bone without suturing (sheet group). The transplanted osteogenic matrix cell sheets filled the gap between the grafted bone and host bone (Figure 2). The control group consisted of grafted bone without an osteogenic matrix cell sheet. Unprotected weight bearing was allowed immediately after the operation.

### 2.6. Evaluation of Bone Formation

**Figure 3:**
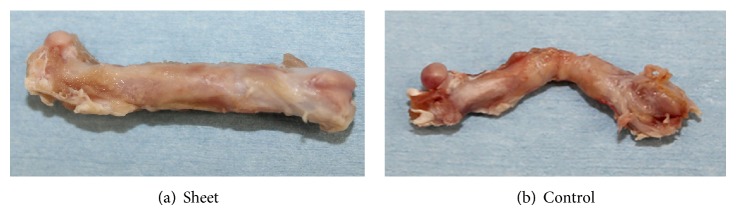
Microscopic appearance of the harvested femur at 12 weeks postoperatively. (a) Sheet group shows that smooth and rigid callus covers the grafted bone, resulting in bone union. (b) Control group shows that soft tissue covers the grafted bone, resulting in nonunion.

X-ray images were taken under anesthesia at 4, 8, and 12 weeks postoperatively to evaluate bridging bone formation around the grafted devitalized bone. Rats were sacrificed and right hind limbs were harvested at 12 weeks postoperatively (Figure 3). After removal of the Kirschner wires, micro-computed tomography (CT) images were recorded using a Microfocus X-ray CT system. Quantitative 3D analysis was performed using a micro-CT system (SMX-160CTS; Shimadzu Corporation, Kyoto, Japan).

Two of the harvested femurs in each group were dissected from the surrounding muscle, fixed in 10% formalin, and embedded in methyl methacrylate resin. For histology, the femurs were cut longitudinally and subjected to von Kossa and Villanueva-Goldner staining. In von Kossa staining, calcified bone is represented as black. In Villanueva-Goldner staining, calcified bone is represented as green and an osteoid is represented as red.

**Figure 4:**
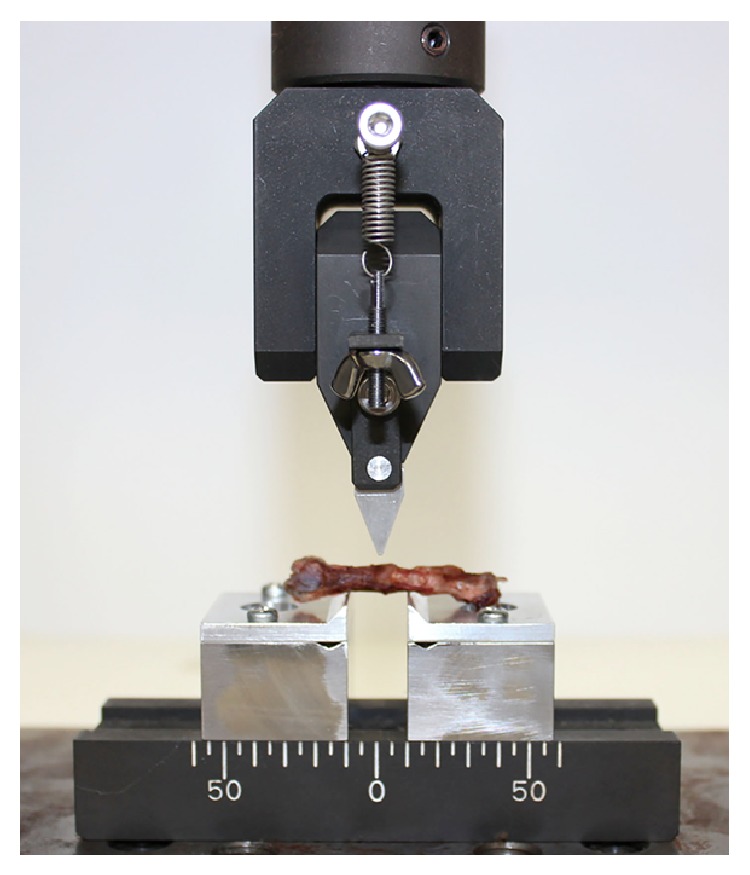
Three-point bending test.

Eight of the harvested femurs in each group were applied to mechanical testing (three-point bending test) using a universal testing machine equipped with a computer for data acquisition (EZ Graph; Shimadzu Corporation). As shown in Figure 4, a load was placed on the junction between the grafted bone and host femur. A crosshead speed of 10 mm/minute was applied until rupture. The maximum bending load at rupture was considered as the failure force.

### 2.7. Statistical Analysis

Statistically significant differences in the mechanical test analyses were determined by the Mann-Whitney *U* test. A value of *P* < 0.01 was considered to be statistically significant.

## 3. Results

### 3.1. X-Ray Images

**Figure 5:**
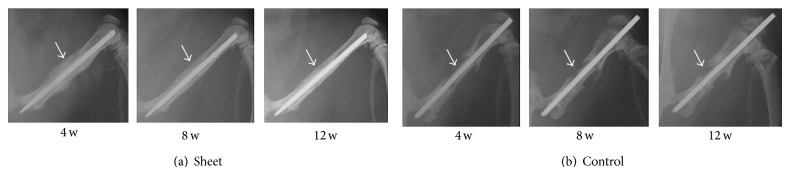
X-ray images of bone-grafted sites (femur) at 4, 8, and 12 weeks postoperatively. Arrows indicate bone-grafted sites. (a) Sheet group shows callus formation around the bone-grafted site, resulting in bone union at 12 weeks. (b) Control group shows nonunion.

X-ray images taken at 4 weeks after transplantation showed obvious callus formation around the grafted bone in the sheet group. Callus formation had developed during the experimental period and bridging bone formation with the host bone was observed at 12 weeks postoperatively, resulting in bone union. In contrast, the control group showed no bridging callus formation around the grafted bone even at 12 weeks (Figure 5).

### 3.2. CT Images

**Figure 6:**
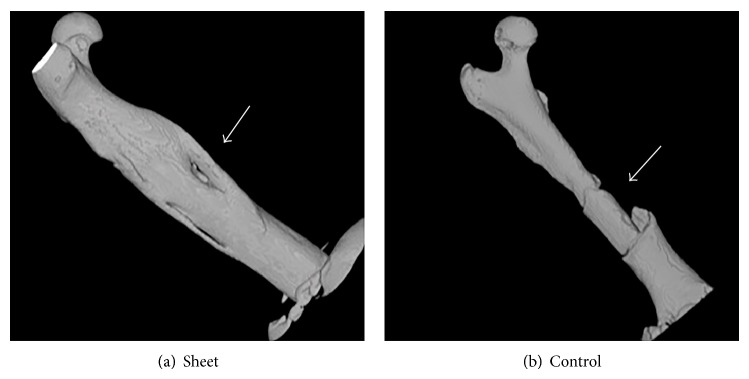
Micro-CT images at 12 weeks postoperatively. Arrows indicate bone-grafted sites. (a) Sheet group shows bridging callus formation covering the grafted bone. (b) Control group does not show callus formation around the grafted bone.

Micro-CT images recorded at 12 weeks postoperatively showed bridging bone formation around the grafted bone in the sheet group. In contrast, the control group exhibited no bridging bone formation around the grafted bone even at 12 weeks (Figure 6), which was similar to the X-ray images at 12 weeks.

### 3.3. Histological Staining

**Figure 7:**
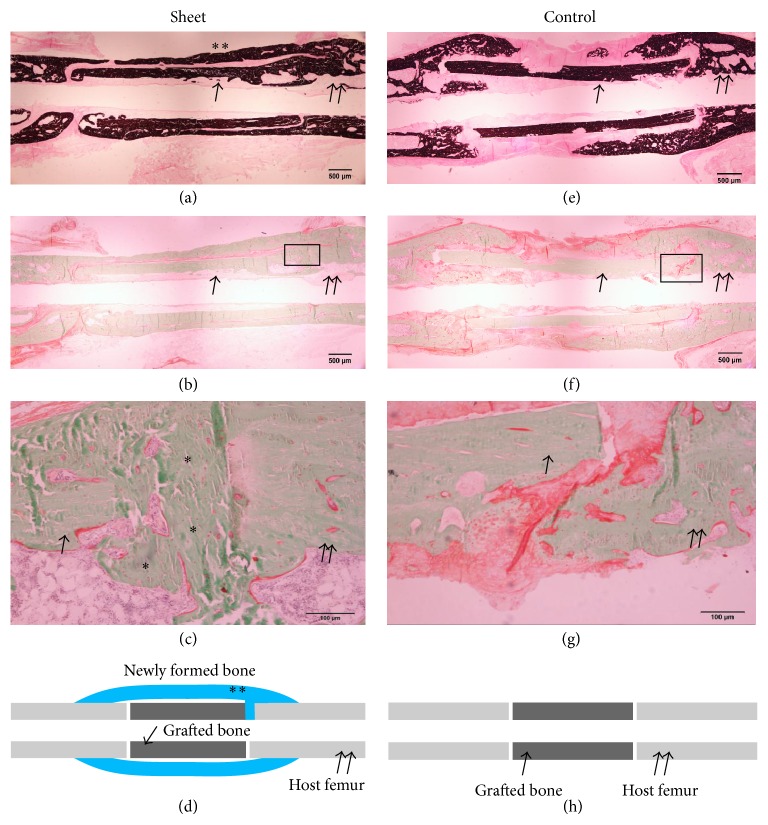
Histology of the harvested femur at 12 weeks postoperatively. Single and double arrows indicate grafted bone (irradiated bone) and the host femur, respectively. Asterisks indicate newly formed bone. (a), (b), (c), and (d) Sheet group; (e), (f), (g), and (h) control group. (a) and (e) von Kossa staining; (b), (c), (f), and (g) Villanueva-Goldner staining. (a) New bone formation was observed around the grafted bone. (b) New bone formation was also observed around the grafted bone. (c) Higher-magnification image of the rectangular area in (b). Grafted bone and the host femur were united by newly formed bone (asterisks). (d) Diagram of (a) and (b). (e) No bone formation was observed around the grafted bone. (f) Bone formation was also not observed around the grafted bone. (g) Higher-magnification image of the rectangular area in (f). Fibrous tissue was observed between the grafted bone and host femur, indicating nonunion. (h) Diagram of (e) and (f).

Representative histological sections of the harvested femur showed new bone formation around the grafted bone in the sheet group. In Villanueva-Goldner stained sections, grafted bone with empty lacunae and the host femur were united by the newly formed bone. Although there was no obvious remodeling at 12 weeks postoperatively, the grafted bone appeared to be stabilized by the newly formed bone in the gap between grafted and host bones as well as around the whole circumference of the graft. In contrast, no bone formation was observed around the grafted bone in the control group. Soft tissue interposition was observed between grafted and host bones, which was consistent with the nonunion findings (Figure 7).

### 3.4. Mechanical Testing

In the three-point bending test, the median failure forces at the site of the junction between the grafted bone and host femur were 101.2 N (41.7–233.5) and 14.2 N (0−70.3) in the sheet and control groups, respectively.

**Figure 8:**
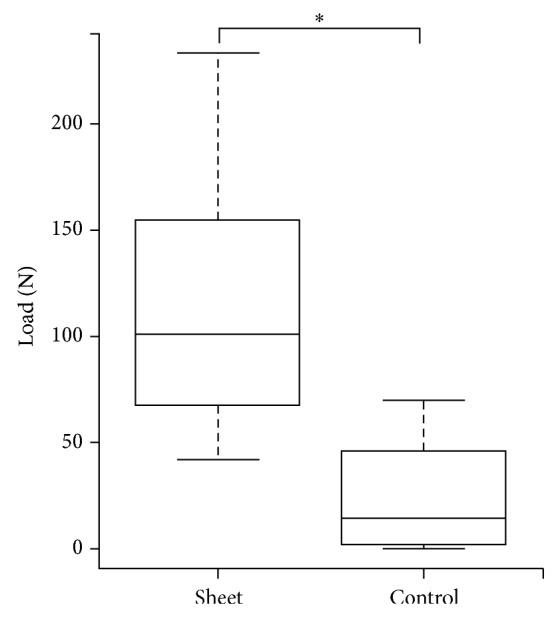
Results of mechanical testing (three-point bending test). Failure force in the sheet group was significantly higher than that in the control group (^∗^ *P* < 0.01).

The failure force in the sheet group was significantly higher than that in the control group (*P* = 0.004) (Figure 8).

## 4. Discussion

The present study clearly demonstrated that osteogenic matrix cell sheets can facilitate the osteogenesis of autogenous irradiated devitalized bone grafts. By combining the devitalized bone graft with an osteogenic matrix cell sheet, bone union was achieved at 12 weeks postoperatively. Although remodeling of the bone graft was not observed until 12 weeks, we observed bridging bone formation over the grafted bone between both ends of the autogenous bone, resulting in stability between the grafted bone and host bones. Mechanical testing revealed significantly higher strength in the sheet group, indicating that transplantation of devitalized bone grafts with osteogenic matrix cell sheets can be useful for bone reconstruction after musculoskeletal tumor resection.

In clinical cases of bone defects after musculoskeletal tumor resection, intraoperative extracorporeal autogenous irradiated bone grafting is one of the bone reconstruction methods. The method consists of a wide en bloc resection of the tumor, curettage of the tumor, extracorporeal irradiation with 50 Gy, and reintroduction of the irradiated bone into the host with fixation devices. The problem with this reconstruction method is that the osteogenic capacity is lost by irradiation and nonunion or delayed union often occurs [5, 8]. Therefore, an extended treatment strategy is required for intraoperative extracorporeal autogenous irradiated bone grafting. The present study indicates that transplantation of an osteogenic matrix cell sheet may be a strategy to solve this problem.

BMSCs have a self-renewal potential and can differentiate into osteoblasts, adipocytes, chondrocytes, neurons, and myogenic cells [12, 13, 15]. Differentiation into the osteoblastic lineage is induced by* in vitro* culture in osteoinductive medium containing Dex, Asap, and *β*-glycerophosphate [16–18]. BMSCs have been widely applied to a variety of scaffolds as a cell source for tissue regeneration including bone and cartilage reconstruction [16, 19, 20]. However, a scaffold that has no biological activity may function as a barrier for bone regeneration [9]. Recently, we reported that BMSCs can be transplanted as a cell sheet (osteogenic matrix cell sheet) with an osteogenic potential [9–11]. Our cell sheet transplantation method does not require specialized equipment to create the cell sheet, because the BMSCs are simply cultured in osteoinductive medium and lifted as a cell sheet using a scraper. Osteogenic matrix cell sheets remain at the transplantation site and fill the gap between the grafted bone and host bone (as shown in Figure 2), even when they are freely transplanted onto a scaffold at a fracture site. In our previous study, we analyzed the origin of* de novo* formed bone in the transplantation model of osteogenic matrix cell sheets using the sex-determining region Y (*Sry*) gene as a marker of donor cells.* Sry* was detected in the* de novo* formed bone, indicating that the cells in the transplanted osteogenic matrix cell sheet without a scaffold could survive at the fracture site and differentiate into osteogenic lineage cells* in vivo* [11].

We used irradiated devitalized bone as a graft in the present study. Therefore, the outcome of the graft and host bones might easily be nonunion because of the interposition of soft tissue in the junctions between them. However, we observed bridging bone formation over the grafted bone between both ends of the host bones as well as* de novo *formed bone in the junction. This union may be achieved by the fact that the osteogenic matrix cell sheets are pulpy and able to enter the gaps, that is, bone fracture sites, nonunion sites, and bone-grafted sites. Cell sheet transplantation prevented interposition of soft tissue into the gaps between the graft and host bones. Subsequently, bone tissue was formed in the gap as well as around the whole circumference of the graft. Therefore, the present study indicates that transplantation of an osteogenic matrix cell sheet provides an osteoinductive matrix as well as osteogenic cells, resulting in facilitation of osteogenesis.

Moreover, the osteogenic matrix cell sheet produces angiogenic factors, such as vascular endothelial growth factor A (VEGF-A), thereby promoting vessel sprouting from the existing vasculature and blood flow around the devitalized bone. Osteogenic matrix cell sheet transplantation can be applied to not only bone fractures but also nonunion and necrotic bone sites. Therefore, we believe that cell sheet transplantation can contribute to bone reconstruction in cases involving not only nonunion but also irradiated bone grafts.

The present study has some limitations. First, we used an irradiated bone model for our analyses. Clinically, intraoperative extracorporeal bone devitalized with liquid nitrogen is also used for reconstruction of bone defects after resection of malignant musculoskeletal tumors, because devitalization by liquid nitrogen can preserve the cartilage matrix and sufficient biomechanical strength [21–23]. Here, we chose irradiation that causes denaturation of proteins. Nevertheless, our transplantation method united irradiated bones to the host bones, indicating a high osteogenic activity. Second, the follow-up duration of 12 weeks was relatively short in the present study. Histological sections of the harvested femur showed little remodeling in the irradiated bone. Without remodeling, grafted bone and the junctions may be weak and subsequently fracture. However, in clinical cases, an intramedullary nail or plate can provide stability and prevent fractures at the site of the grafted bone and the junctions between the grafted bones and host bones until completion of union and remodeling. In a long-term followup, remodeling might occur during the healing process of the fracture. Further study is needed to address these points. Third, we did not evaluate vascularization around the irradiated bone. Revascularization is an important factor for new bone formation around irradiated devitalized bone. Angiogenic factors such as VEGF-A in the osteogenic matrix cell sheet may promote revascularization and vascular flow. Finally, we need to extend our study by performing experiments with larger bone defects and different species.

## 5. Conclusions

Our study clearly indicates that osteogenic matrix cell sheet transplantation facilitates osteogenesis in irradiated devitalized bone grafts. Therefore, transplantation of autogenous irradiated bone grafts with osteogenic matrix cell sheets may become a treatment option for reconstruction of bone defects after resection of malignant musculoskeletal tumors.
